# Markerless 2D/3D image guidance vs. 2D fluoroscopy in posterior pedicle screw fixation using intraoperative 2D imaging: An experimental study

**DOI:** 10.1016/j.bas.2025.104395

**Published:** 2025-08-27

**Authors:** Benno Bullert, Eric Mandelka, Paul A. Gruetzner, Sven Y. Vetter, Jula Gierse

**Affiliations:** BG Klinik Ludwigshafen, Department for Orthopedics and Trauma Surgery at Heidelberg University, Ludwigshafen, Germany

**Keywords:** Minimally invasive surgical procedures, Spinal fusion, Pedicle screws, Image-guided, Computer-assisted, Intraoperative imaging

## Abstract

**Introduction:**

Posterior fixation with pedicle screws (PS) is a standard procedure in spine surgery. While 3D navigation enhances accuracy and minimizes radiation exposure, the high technical demands and costs typically restrict its use to specialized centers. A potential alternative is markerless 2D/3D image guidance, which enables precise 3D guidance using a standard 2D C-arm.

**Research question:**

This experimental study aims to determine whether 2D/3D image guidance enhances accuracy, speed, and radiologic parameters in thoracic and lumbar PS placement compared to 2D fluoroscopy.

**Material and methods:**

This experimental study used radiopaque artificial spine models to compare posterior PS placement between 2D/3D image guidance and 2D fluoroscopy. Three surgeons with different experiences in spine surgery placed 120 PS in total. Two raters assessed accuracy using the Gertzbein-Robbins grading system (GRS).

**Results:**

K-wire placement accuracy was higher with 2D/3D image guidance (*p = 0.012*). PS placement accuracy (GRS A/B) was significantly higher with 2D/3D image guidance (96.7 %) than with 2D fluoroscopy (80.0 %, *p = 0.005*). Average PS insertion time was longer with 2D/3D image guidance (7.12 ± 2.42 min) than with 2D fluoroscopy (2.89 ± 1.29 min, *p < 0.001*).

**Discussion and conclusion:**

Markerless 2D/3D image guidance significantly enhances the accuracy of PS placement compared to 2D fluoroscopy, although it requires more time for each placement. This system has the potential to improve precision in minimally invasive spine surgery, especially in settings without access to advanced 3D navigation systems. However, the results are based on artificial bone models and limited user experience, which may reduce their generalizability to clinical practice.

## Highlights

• Markerless 2D/3D image guidance achieved higher pedicle screw accuracy than 2D fluoroscopy.
• Inexperienced spine surgeons achieved high accuracy with markerless 2D/3D image guidance.
• Markerless 2D/3D image guidance offers 3D guidance without intraoperative 3D imaging.
• Markerless 2D/3D image guidance offers 3D guidance with a standard 2D C-arm.

## 1 Introduction

Posterior fixation with pedicle screws (PS) is a standard procedure in spine surgery. Minimally invasive, percutaneous techniques are used more frequently, reducing operating time, blood loss, and hospitalization time (McAnany et al., 2016; Mobbs et al., 2012).

Percutaneous PS placement can be controlled using 2D fluoroscopy or 3D navigation. The comparison between these techniques shows that the use of 3D navigation can achieve higher screw placement accuracy and, at the same time, produce lower radiation exposure for the operating room (OR) staff (Mason et al., 2014; Mandelka et al., 2023). The high acquisition costs of navigation systems and intraoperative 3D imaging restrict the availability of 3D navigation primarily to specialized high-volume centers in highly developed countries. Therefore, most percutaneous posterior fixations are still performed using 2D fluoroscopy or referencing anatomical landmarks (Härtl et al., 2013).

A promising alternative to conventional 2D techniques is real-time markerless 2D/3D image guidance, which combines intraoperative 2D fluoroscopy with preoperative CT or intraoperative cone beam CT (CBCT) through advanced registration algorithms. These algorithms align intraoperative 2D X-ray images with a preoperative CT scan or an intraoperative CBCT, ensuring that the 2D X-ray and 3D dataset share the same coordinate system (Miao et al., 2016).

In contrast to traditional fluoroscopy-based navigation systems, which typically require dedicated optical cameras and surgical instruments equipped with tracking markers, or surface matching techniques that involve fixed patient-mounted reference arrays and additional calibration steps, markerless 2D/3D guidance requires no specialized hardware, no optical tracking, and no dedicated instruments. This enables precise and real-time image-based instrument guidance using standard intraoperative 2D fluoroscopy and conventional surgical tools, without the need for tracking markers or specialized equipment. The approach can be integrated into existing operating room workflows and may be particularly valuable in settings where conventional navigation systems are not available.

Previous studies on 2D/3D image guidance have primarily focused on improving accuracy, computational efficiency, and the application of various algorithms (Uneri et al., 2017; Mi et al., 2023; Unberath et al., 2021). However, to the authors’ knowledge, no prior evaluation has been conducted on a 2D/3D image guidance system that combines real-time computational performance suitable for intraoperative use with integrated tool detection and a user interface designed to be operable by first-time users.

This study aims to compare the performance of minimally invasive posterior fixation of the thoracic and lumbar spine with pedicle screws using intraoperative 2D imaging only. For this purpose, we compare a new markerless 2D/3D image guidance technique versus conventional 2D fluoroscopy in terms of accuracy, time, imaging parameters, and the surgeon's experience.

We hypothesize that markerless 2D/3D image guidance will improve pedicle screw placement accuracy compared to conventional 2D fluoroscopy, regardless of the surgeon's experience. Procedural time and imaging-related parameters are assessed in an exploratory manner.

## 2 Material and Methods

In this experimental study, PS placement was performed on six identical radiopaque artificial spine models (Synbone, Zizers, Switzerland). A comparison between 2D fluoroscopy (C-arm: Cios Spin, Siemens Healthineers AG, Erlangen, Germany) and the combination of C-arm (Cios Spin, Siemens Healthineers AG, Erlangen, Germany) and a 2D/3D image guidance software (VUZE, Vuze Medical, Ra'anana, Israel) was performed.

PS were placed by three different surgeons: an experienced spine surgeon (ES) familiar with spinal navigation, a third-year ‘experienced resident’ (ER) with experience in spine surgery, and a first-year ‘unexperienced resident’ (UR) with minimal experience in spine surgery.

60 PS were placed per imaging technique, resulting in 40 PS per surgeon and 20 PS per surgeon and imaging technique. The PS system used in this study was Reline MAS (NuVasive Inc., San Diego, California, USA).

Due to the lack of preliminary data, a formal sample size calculation was not feasible. The number of pedicle screws per surgeon was therefore aligned with peer-reviewed studies comparing novel navigation techniques to standard procedures in spine surgery (Vaccaro et al., 2020; Müller et al., 2020; Hahn et al., 2015).

### Ethical approval

The study was approved by the responsible ethics committee of the state medical association (application number: 2024–17616). All procedures were in accordance with the ethical standards of the institutional and national research committee and with the 1964 Helsinki Declaration and its later amendments or comparable ethical standards.

### 2.1 Setup

The C-arm's dose setting was set to ‘standard’, and the acquisition mode was set to ‘fluoroscopy’ throughout the procedure. The pulse rate was set to 15 Hertz (Hz). Due to the implementation of the 2D/3D image guidance, the software could be displayed on the right monitor of the C-arms’ monitor cart.

The 2D/3D registration software enables real-time 2D/3D registration and image guidance using intraoperative 2D X-rays combined with preoperative or intraoperative 3D datasets (CT/CBCT). Additionally, instruments are detected in each 2D X-ray image and merged with the preoperative 3D dataset. This functionality facilitates intraoperative image guidance based on 2D X-rays (2D/3D image guidance) without the need for a marker-based external navigation system. The workflow is structured into three main steps: planning, K-wire placement, and PS insertion.

The 2D/3D registration software was executed on a notebook connected to the C-arm via a single network cable. The software interface was displayed on the monitor of the C-arm, another external monitor, and the mobile touch interface.

### 2.2 Procedure

Before data collection, the surgeons had already gained between 2 and 4 h of experience with the 2D/3D image guidance. This included a 1-h theoretical introduction followed by expert-supervised placement of four pedicle screws in the lumbar spine using the same artificial bone model as in the study.

Before the procedure, trajectories were planned by the surgeon, marking three key points for each PS (skin incision point, bone entry point, and screw tip) and one additional key anatomic landmark for each vertebra (spinous process).

The optimal skin incision region was first located during the procedure by positioning a Jamshidi needle or scalpel on the skin, which was visualized in an X-ray image. After the skin incision, the Jamshidi needle was aligned with the bone entry point. If the trajectory was sufficiently adjusted, a 3D reconstruction was generated using a second X-ray (2-image 3D reconstruction) from an angle suggested by the software (range: ±20° angulation and ±10° orbital rotation from the 0° anterior-posterior (AP) C-arm position). Adjustments to the trajectory required a new 3D reconstruction with two additional X-rays. Once confirmed, the Jamshidi needle was advanced, and further 3D depth updates were performed with a single X-ray (1-image 3D reconstruction). Should a trajectory change occur during this time, two additional X-rays were taken, and the 3D position was updated.

**Fig. 1:**
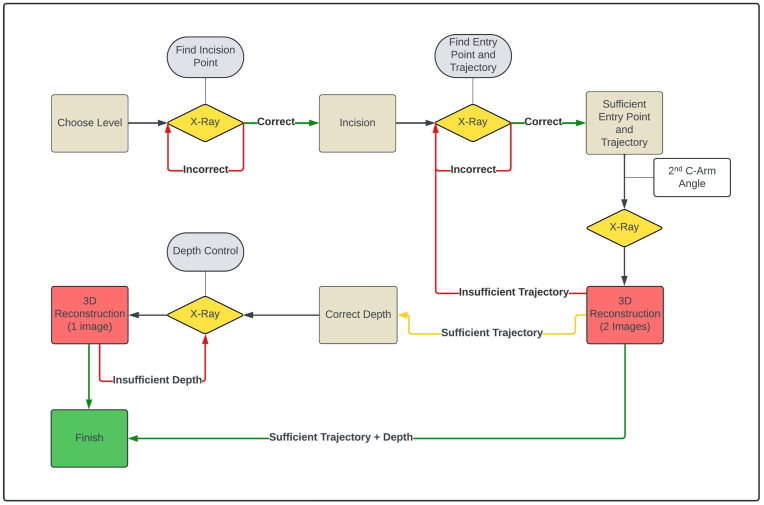
Workflow of 2D/3D Image Guidance Schematic illustration of the procedural steps using markerless 2D/3D image guidance, including imaging, trajectory planning, and verification via 3D reconstruction.

A K-wire was inserted through the Jamshidi needle, and the appropriate PS dimension was determined. Although the software does not officially support screw detection, 2D/3D registration enables screw depth control. Cannulated PS was inserted over the K-wire, and its depth was assessed using a 3D reconstruction from a single AP X-ray. Significant deviation between PS and K-wire trajectories necessitated a 2-image 3D reconstruction for further depth updates, concurrent to trajectory changes of the Jamshidi needle during insertion (Fig. 1).

In minimally invasive 2D fluoroscopy, the vertebral body to be instrumented was first positioned correctly in the beam path by angulation of the C-arm so that the pedicles could be identified. The incision was then made, and the Jamshidi needle was positioned under fluoroscopic control. If the positioning was correct, the Jamshidi needle was advanced and replaced by a wire. The PS (previously measured in the preoperative CT data set) was then inserted over the wire under fluoroscopic control with lateral X-ray images.

### 2.3 Parameters

The positions of the K-wires and PS were evaluated independently by two raters using CBCT 3D datasets. The K-wire position was categorized into In (within the pedicle cortex) and Out (outside the pedicle cortex). For PS, measurements were taken at the narrowest section of the pedicle. The accuracy of screw placement was assessed according to the Gertzbein-Robbins grading system (GRS), where Grades A (no breach) and B (breach <2 mm) were classified as ‘accurate’. Grades C through E (breach ≥2 mm) were deemed potentially critical and ‘inaccurate’. In cases of disagreement between the two raters due to variations in measured values, a re-evaluation was performed to reach a consensus.

PS insertion time was calculated by adding up the time required to insert the K-wire (first X-ray until the K-wire was in a satisfactory position) and the time needed to insert the PS (handing the screwdriver until the screw was in a satisfactory position).

### 2.4 Statistical analysis

Statistical analyses were performed using IBM SPSS Statistics Version 29 (IBM Corp., Armonk, NY, USA). Quantitative data are expressed as mean ± standard deviation (95 % CI). Kolmogorov-Smirnov test was used to test the data for normal distribution. Unpaired, two-level data were analyzed using a *t*-test (normally distributed) or Mann-Whitney-test (non-normally distributed). Unpaired, multi-level data were analyzed using one-way ANOVA (normally distributed) or Kruskal-Wallis-test (non-normally distributed). A significance level of α < 0.05 was set. In the pairwise comparison with multiple testing, α was adjusted using Bonferroni correction. Inter-rater reliability was determined using Cohen's Kappa and assessed according to Landis and Koch's suggestion (Landis and Koch, 1977).

## 3 Results

A total of 120 pedicle screws were placed and analyzed in this study. Therefore, for each imaging technique, 60 pedicle screws were placed.

### 3.1 Accuracy

During K-wire placement, significantly more wires were positioned correctly within the pedicle using 2D/3D image guidance (100 % accuracy), whereas six wires were placed outside the pedicle with 2D fluoroscopy (90 % accuracy) (*p = 0.012*).

**Fig. 2:**
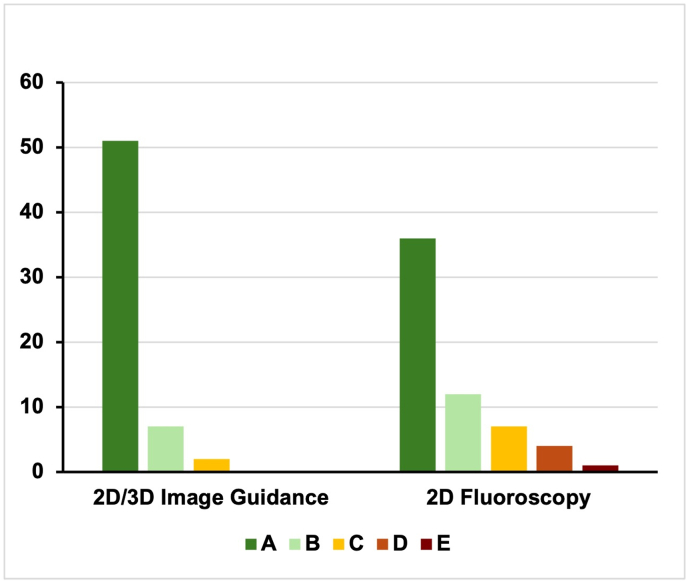
Accuracy Rating of Pedicle Screws by Gertzbein-Robbins Scale (GRS) Grouped GRS classifications (A–E) for screws placed with 2D/3D image guidance and 2D fluoroscopy.

The percentage of correctly placed pedicle screws (GRS A/B) was significantly higher with 2D/3D image guidance (96.7 %) than with 2D fluoroscopy (80.0 %; *p = 0.005*; Fig. 2).

Severe malpositions (GRS D/E) with complete screw diameter outside the pedicle only occurred with 2D fluoroscopy (n = 5; 8.33 %).

There was no significant difference in accuracy between unexperienced resident surgeon (UR), experienced resident surgeon (ER), and experienced spine surgeon (ES) (*p = 0.923*; Table 1).

**Table 1:** Pedicle screw accuracy between the different surgeons.

Surgeon	2D/3D Image Guidance		2D Fluoroscopy		Comparison
	Accurate/Inaccurate	Accuracy	Accurate/Inaccurate	Accuracy	Δ Accuracy
UR	19/1	95.0 %	16/4	80.0 %	+15.0 %
ER	19/1	95.0 %	16/4	80.0 %	+15.0 %
ES	20/0	100.0 %	16/4	80.0 %	+20.0 %

The interrater reliability after the initial rating of PS placement was substantial (κ = 0.655; *p < 0.001*). Re-evaluation and reaching consensus were performed in n = 19 PS.

### 3.2 Time

**Fig. 3:**
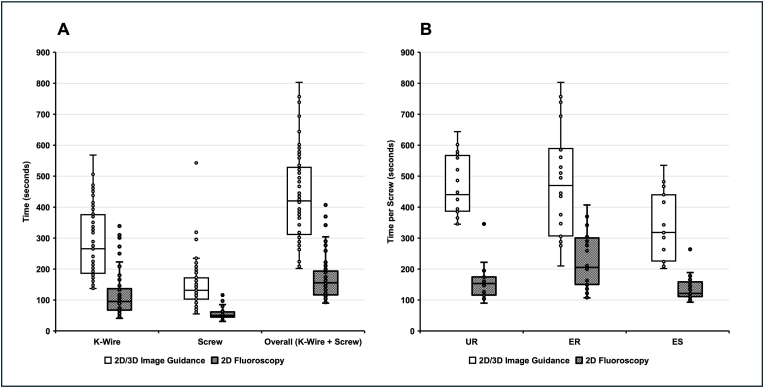
Procedural Time per Pedicle Screw (A) Time required for K-wire, screw, and overall placement using both imaging techniques. (B) Time per screw stratified by surgeon experience (UR, ER, ES) for each technique.

With 2D/3D image guidance, the surgeons required an average of 4.70 ± 1.83 minutes (min) to place the K-wire and 2.42 ± 1.27 min to place the PS. This corresponds to an average of 7.12 ± 2.42 min (K-wire + PS). With 2D fluoroscopy, the surgeons required significantly less time on average for the placement of the K-wire (1.97 ± 1.21 min; *p < 0.001*) and the PS (0.92 ± 0.27 min; P < 0.001), which corresponds to a total time of 2.89 ± 1.29 min (*p < 0.001*; Fig. 3A).

The experienced surgeon (ES) performed the instrumentation with 2D/3D image guidance significantly faster than the experienced resident (ER)(*p = 0.004*) and the unexperienced resident (UR)(*p = 0.002*). However, in 2D fluoroscopy, the ER was significantly slower than the other two surgeons (Fig. 3B).

### 3.3 Radiological parameters

**Fig. 4:**
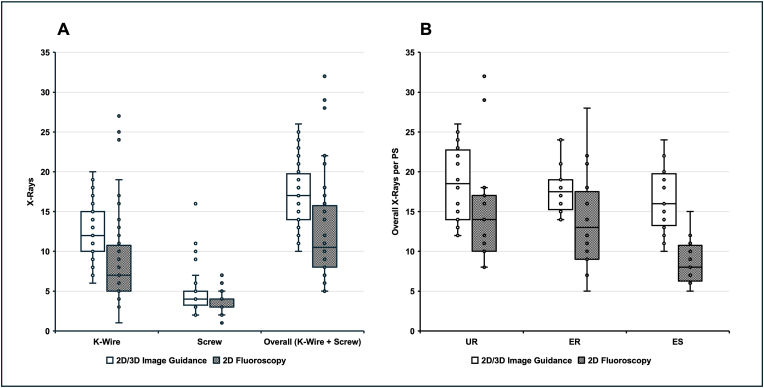
Number of X-rays per Pedicle Screw (A) Number of X-rays acquired during K-wire and screw placement per technique. (B) X-ray usage per screw by surgeon experience and imaging method.

With 2D/3D image guidance, the surgeons required significantly more X-rays than with 2D fluoroscopy (17.43 ± 3.93 vs. 12.30 ± 5.87; *p < 0.001*; Fig. 4A). The average fluoroscopy time (fluoro-time) was 0.90 ± 0.13 seconds (s) per X-ray. A significant difference was found between the individual surgical steps when placing the K-wire (*p < 0.001*) and when placing the pedicle screw (*p = 0.005*).

The number of X-rays between the operators showed no significant differences in the 2D/3D image guidance (*p = 0.271*). However, there were significant differences in 2D fluoroscopy, with fewer X-rays for the ES (*p = 0.002*; Fig. 4B).

## 4 Discussion

### 4.1 Accuracy

This study shows that 2D/3D image guidance enables significantly higher accuracy in K-wire placement compared to conventional 2D fluoroscopy. For pedicle screws, 2D/3D image guidance achieved an accuracy of 96.7 % compared to 80.0 % with 2D fluoroscopy, corresponding to a relative improvement of 20.8 %. This was shown equally for both experienced and unexperienced spine surgeons.

The comparison of accuracy between different studies in the literature is difficult due to the different classifications. A systematic review of 43,305 PS by Aoude et al. used a grading comparable to this study (GRS A + B; breach <2 mm) and showed an accuracy of 91.4 % with free-hand or 2D fluoroscopy and an accuracy of 97.3 % for navigated procedures (Aoude et al., 2015).

The differences between the literature and this study regarding the accuracy of 2D fluoroscopy are presumably due to the use of artificial bone, which has certain disadvantages for the surgeon, particularly in haptic feedback, making instrumentation more challenging. From this perspective, however, it is even more remarkable that the accuracy of 2D/3D image guidance is on par with that of 3D navigated or robotic procedures.

Studies and grading systems for the position of PS usually focus solely on the risk of neurovascular damage and sometimes assume wide perforations of up to 4 mm as a ‘safe zone’ (Perdomo et al., 2019). With an increasingly aging patient population and compromised bone quality, the biomechanical aspect of PS placement should not be neglected. A lateral PS perforation of 2 mm or 4 mm already reduces biomechanical stability by 27.8 % and 50.9 %, respectively, in the lumbar spine (Tsagkaris et al., 2024). It should, therefore, be avoided in any case. Looking at the subgroup of accurate PS in this study (GRS A/B), using 2D/3D image guidance shows a higher proportion of PS in the optimal position without pedicle breach (GRS A). Thus, PS placed with 2D/3D image guidance are more accurate and, therefore, should show a lower risk of neurovascular complications and should also potentially be biomechanically superior.

### 4.2 Time

Pedicle screw placement using 2D fluoroscopy was significantly faster than using 2D/3D image guidance for all surgeons, requiring only about 41 % of the time needed for the 2D/3D image-guided technique. A comparison of the surgeons shows a significantly faster PS placement for experienced spine surgeon (ES) compared to the resident surgeons (UR; ER) using 2D/3D image guidance.

Studies on the insertion time per PS with 2D fluoroscopy show 6.2–7.6 min/PS (Vaccaro et al., 2020; Müller et al., 2020; Hahn et al., 2015). With 3D navigation, a study by Ryang et al. showed a time of 5.3 min/PS placement (Landis and Koch, 1977). Studies on robot-assisted PS placement show times of 3.5–7.8 min/PS (Müller et al., 2020; Aoude et al., 2015).

Studies have demonstrated a significant learning curve regarding time as a parameter for both navigated and robotic procedures (Jiang et al., 2023; Ryang et al., 2015). Therefore, it can be assumed that the time required per pedicle screw placement with the new 2D/3D image guidance system serves merely as an indication and does not yet provide definitive conclusions about the actual intraoperative time requirements. Nevertheless, compared to times reported in the literature, the 2D/3D image guidance system is similar to established methods. In contrast, 2D fluoroscopy demonstrates significantly shorter times in this study compared to times reported in the literature.

While enabling faster pedicle screw placement, 2D fluoroscopy was associated with lower accuracy compared to 2D/3D image guidance. This highlights a relevant trade-off between procedural speed and placement precision. Secondary factors, such as a potential reduction in revision surgeries, as demonstrated in conventional 3D navigation systems (Suri et al., 2025), and improved efficiency with increasing user experience, may influence the overall procedural time in clinical practice. However, this requires confirmation in future clinical studies.

### 4.3 Radiological parameters

Using 2D/3D image guidance, surgeons required more X-rays per PS placement than with 2D fluoroscopy, corresponding to a 41.7 % increase. A comparison between the surgeons shows they needed similar images per PS placement regardless of their experience with the new 2D/3D image guidance. With 2D fluoroscopy, however, the experienced surgeon required significantly fewer images than the two residents.

The mean fluoro-time per PS using 2D fluoroscopy is between 17.8 and 29.0 s (Jamshidi et al., 2021; Laine et al., 2000; Mroz et al., 2011) in the literature. Based on the number of X-rays, the average fluoro-time was 15.7 ± 3.5 s per PS for 2D/3D image guidance and 11.1 ± 5.3 s per PS for 2D fluoroscopy. Therefore, both values are lower than the comparative values in the literature, probably due to the experimental setting on the artificial bone. Due to the good visualization of the pedicles on the artificial bone and the lack of real soft tissue, it is presumably possible to find the entry point faster with the Jamshidi needle, which reduces the fluoro-time for 2D fluoroscopy. Due to the higher minimum number of projections for 3D visualization, 2D/3D image guidance cannot equally exploit this advantage on artificial bone, which means that the comparison of this parameter on artificial bone can only be transferred to a clinical setting to a limited extent.

Due to the low scattered radiation emission from artificial bone, it was impossible to measure radiation exposure for the surgeons. Nevertheless, it should be discussed that no lateral images were taken in 2D/3D image guidance. In contrast, in 2D fluoroscopy, at least 30 % of all X-rays (for PS insertion) were taken in the lateral beam path. These lateral images demonstrate up to a 30-fold increase in radiation exposure for the surgeon (Yamashita et al., 2023), highlighting the potential clinical relevance of their elimination through 2D/3D image guidance in terms of reducing radiation exposure. While this suggests that 2D/3D guidance may reduce radiation exposure for the surgical team compared to conventional 2D fluoroscopy, verification under clinical conditions with real scatter radiation is needed to confirm this potential benefit.

### 4.4 Comparison to marker-based 2D/3D techniques

Conventional intraoperative navigation is typically based on marker-based registration, using either active or passive optical markers in combination with optical camera systems (Wilson et al., 2024). 2D/3D registration algorithms are already in clinical use within such systems to register preoperative CT datasets to the patient's anatomy (Vaccaro et al., 2020). This registration is usually performed once at the beginning of the procedure using fixed reference arrays, and all subsequent navigation relies on this initial alignment. Accurate intraoperative instrument tracking then depends on specialized hardware, including optical tracking systems and marker-equipped instruments.

In contrast, the technique evaluated in this study employs a markerless 2D/3D image guidance that performs real-time registration with each new intraoperative X-ray. This approach eliminates the need for external tracking cameras, reference arrays, or specialized marker-equipped instruments. Furthermore, it addresses common limitations of marker-based systems, such as line-of-sight interruptions or reference frame shifts, by continuously re-registering the anatomy (Rahmathulla et al., 2014). This dynamic update mechanism offers potential improvements in robustness and accuracy, particularly in settings where rigid referencing is technically challenging.

### 4.5 Comparison to existing studies in the field of 2D/3D image guidance

Previous studies on 2D/3D image guidance have predominantly focused on algorithm development and technical validation in controlled laboratory environments.

Uneri et al. presented a method for 2D/3D registration of vertebral anatomy using digitally reconstructed radiographs (DRRs), which was evaluated under idealized phantom conditions without surgical instrumentation or workflow integration (Uneri et al., 2017). Similarly, Mi et al. developed a deep learning-based framework for surgical tool tracking and registration; however, their evaluation was limited to synthetic and preclinical data, without interaction with human operators or real-time workflow challenges (Mi et al., 2023). Unberath et al. proposed a markerless 2D/3D registration framework based on anatomical features and CBCT datasets, but likewise focused on algorithmic performance rather than practical surgical application (Unberath et al., 2021).

In contrast, the present study evaluates a clinically applicable markerless 2D/3D image guidance system under realistic surgical conditions, and compares it directly to conventional 2D fluoroscopy using task-relevant metrics. While the technical details of the proprietary system are not disclosed, this study is the first to evaluate a 2D/3D registration algorithm in a setting that closely replicates intraoperative conditions, including surgeon interaction and workflow integration. This represents a key step toward translating the technology from controlled laboratory testing into practical surgical application.

### 4.6 Limitations

As already discussed, the main limitation of this study is the use of artificial bone models, which can only represent clinical reality to a certain extent. One reason is that these models only show a slight difference between hard cortical and soft cancellous bone, resulting in a lack of haptic feedback for the surgeons and forcing them to rely more extensively on the images provided. As a result, both the Jamshidi needle and the PS are not guided through the hard cortical bone in the pedicle, which, in turn, can result in lower accuracy rates. Furthermore, there is a lack of realistic soft tissue and hemorrhage, which may allow faster instrumentation. Another limitation is that no reliable radiation dose data could be collected, as the low radiodensity of the models resulted in unusable dose values due to the limited output resolution of the C-arm system. However, we consider fluoroscopy time and the number of 2D images to be more robust and clinically transferable parameters, as radiation exposure in patients varies greatly and is strongly influenced by individual factors such as body mass index. Therefore, further clinical studies are required to validate the efficacy and clinical value of the technology in real-world settings.

The emitted dose of the C-arm was not analyzed, as this value would represent another value proportional to the number of X-rays for identical artificial bones.

A further limitation is the three surgeons' minimal experience using 2D/3D image guidance. Particularly regarding the time parameters, the data from this study can only be limitedly transferred to everyday practice with the system.

In addition, the subjective nature of screw grading using the Gertzbein-Robbins scale may have introduced a minor degree of variability, despite consensus-based evaluation.

## 5 Conclusion

This study demonstrates that markerless 2D/3D image guidance shows significantly higher accuracy of K-wire and pedicle screw placement compared to 2D fluoroscopy, regardless of the surgeon's experience. While these findings highlight its potential to enhance precision in spine surgery, particularly in settings without access to advanced 3D navigation or intraoperative 3D imaging, clinical studies are needed to validate its efficacy and applicability in patient care.

## Declaration of competing interest

The authors declare the following financial interests/personal relationships which may be considered as potential competing interests: Paul Alfred Gruetzner reports a relationship with Siemens Healthineers AG that includes: consulting or advisory and travel reimbursement. The research group MINTOS had grants/grants pending and technical support from Siemens Healthineers AG (Erlangen, Germany) and Globus Medical Inc. (Audubon, Pennsylvania, USA). The funders had no involvement in the study conceptualization, design, data collection, analysis, nor the decision to publish or the preparation of the manuscript. If there are other authors, they declare that they have no known competing financial interests or personal relationships that could have appeared to influence the work reported in this paper.
